# Efficient X-Ray Source Probe Adjustment of the Intrabeam Intraoperative Radiotherapy System

**DOI:** 10.7759/cureus.105730

**Published:** 2026-03-23

**Authors:** Alberto de la Zerda, Kyle R Padgett, Matthew Studenski, Joseph Both, Levent Sensoy

**Affiliations:** 1 Department of Radiation Oncology, Sylvester Comprehensive Cancer Center, Miller School of Medicine, University of Miami, Miami, USA; 2 Department of Radiation Oncology, Jackson Memorial Hospital, Miami, USA

**Keywords:** intrabeam, intraoperative radiotherapy, iort, kv x-rays, operating room, qa, quality assurance, xrs

## Abstract

Intraoperative radiotherapy (IORT) delivers, at the time of surgery, a large dose of radiation in a single fraction to a target area directly accessible to the surgeon in the operating room (OR), allowing for increased dose to the target and decreased dose to nearby normal tissues. Delivery of IORT to a patient requires that pertinent quality assurance (QA) checks of the system be successfully executed to ensure appropriate treatment. A widely used IORT system presently is the Intrabeam unit (Carl Zeiss Meditec AG, Jena, Germany), an electronic brachytherapy system which uses a compact low-energy X-ray source (XRS) mounted on a semi-robotic arm. To deliver IORT treatments using the Intrabeam system, two initial QA tests must be conducted to ensure that the isotropy and the output of the radiation source are both within tolerance. Whenever the initial isotropy check fails, indicating that the probe of the XRS is misaligned, an additional QA procedure meant to realign the probe becomes mandatory. This latter probe-adjustment procedure involves an interplay between the operator manipulating a special QA device around the XRS probe while the system monitors, continuously and in real time, the extent of misalignment of the probe. With a system-specified tolerance for probe eccentricity to be <0.1 mm, this interplay can result repeatedly in false-negative results due to artifacts unrelated to probe alignment, such as XRS manipulation by the user, potentially causing significant delays in initiation of the IORT treatment. We suggest a modified probe realignment procedure whereby measuring artifacts arising while the user manipulates the QA device are disregarded, and instead only artifact-free misalignment readings, i.e., while the device is not being manipulated, are considered. This modification is meant solely to improve efficiency, is based on workflow experience rather than any related quantitative measurements, and does not involve any changes to system tolerances or QA test acceptance criteria. A more efficient realization of the required set of preoperative QA tests could save significant time for the medical teams involved, as well as reduce the total time a patient is maintained under general anesthesia.

## Introduction

Intraoperative radiotherapy (IORT) allows delivery of a high dose of radiation to the tumor bed of a patient at the time of surgery, following excision of a tumor. Modalities to deliver IORT include low-energy X-rays, electron beams, and high-dose-rate (HDR) after-loader units. IORT is currently utilized to treat lesions at sites in the body, including breast, brain, spine, head and neck, gastrointestinal tract, and skin [1]. In addition to allowing delivery of increased dose to target areas with decreased dose to normal tissues, the IORT modality presents, for indicated cases, advantages compared with external beam radiotherapy (EBRT) by reducing the number of treatment visits to a single day instead of multiple days or weeks as required in a course of EBRT. This may be a significant convenience for the elderly and other patient demographics, such as the suburban population, who require daily transportation to urban treatment facilities. A widely used IORT system is the Intrabeam unit (Carl Zeiss Meditec AG, Jena, Germany) with about 75 units installed in the United States and about 325 in the world, according to the manufacturer (information provided by Zeiss). Various types of applicators are available with the Intrabeam equipment, designed to optimally cover the specific type of target volume to be treated while reducing dose to uninvolved tissues near that target [2]. Applicators designed specifically for different treatment sites are available with Intrabeam to obtain appropriate dose distributions, including: “spherical,” for breast lumpectomy and brain lesions; “needle,” for spine or brain lesions; “flat,” for gastrointestinal tract lesions; and “surface,” for skin lesions. Utilization of the Intrabeam system to treat any of these anatomical sites requires completion of strict pre-operative quality assurance (QA) checks of the X-ray source (XRS). A challenging situation can arise in the operating room (OR) when the probe of the XRS is found to be misaligned. Having the source of the X-rays located at the tip of the probe, any deviations of the probe tip larger than the manufacturer's established threshold of <0.1 mm could result in deleterious changes to the dose distribution delivered to the targeted volume, due to the very rapid dose falloff of the low-energy 50 kV X-rays in tissue. Based on our own experience, the necessary process of realigning the probe can introduce significant delays in starting an appropriate IORT delivery, and any efforts in improving efficiency would be beneficial to the IORT team and to a patient maintained under general anesthesia throughout the surgical and radio-therapeutic interventions. After a general introduction to the IORT modality, we detail the rationale and methodologies of the QA pre-operative checks specific to the Intrabeam system and suggest a modification to the probe realignment procedure, intended to improve workflow efficiency of this modality without altering system acceptance criteria.

## Technical report

IORT with the low-energy X-ray Intrabeam system

**Figure 1:**
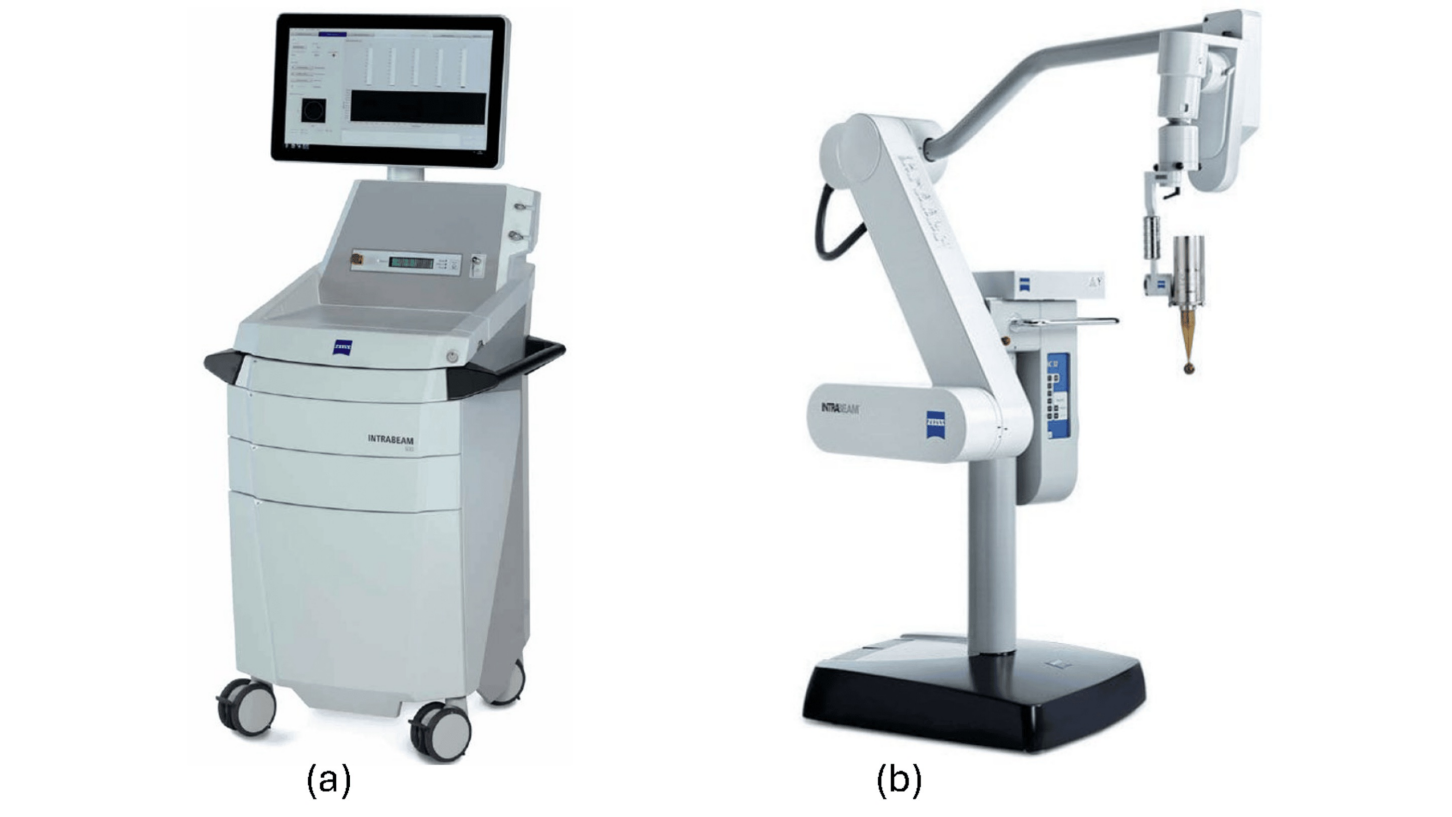
a) The control console cart powers and controls the XRS. b) Semi-robotic arm with an XRS mounted at its end having a spherical applicator attached. The console continuously monitors and records details of treatment delivery. The working area in the front of the console is customized for conducting the daily QA tests. The balanced semi-robotic arm allows the surgeon to manipulate the XRS-applicator assembly into the position and angulation selected for treatment (Figures from Zeiss [3] with permission).

The main components of the Intrabeam system are the control console cart, the semi-robotic arm, and the X-ray source (XRS). The control console (Figure 1a) powers and controls the XRS during treatment and displays details of the treatment delivery in real-time. The computer in the console records treatment details throughout the IORT delivery for documentation and reporting. The QA checks of the XRS are performed on the work area of the cart. The semi-robotic arm (Figure 1b) allows the surgeon to manipulate the XRS into position in the patient for treatment. The arm is supported by a floor stand on wheels, which can be locked in place for the X-ray delivery. The semi-robotic arm is shown with an XRS mounted at its end with an attached spherical applicator.

**Figure 2:**
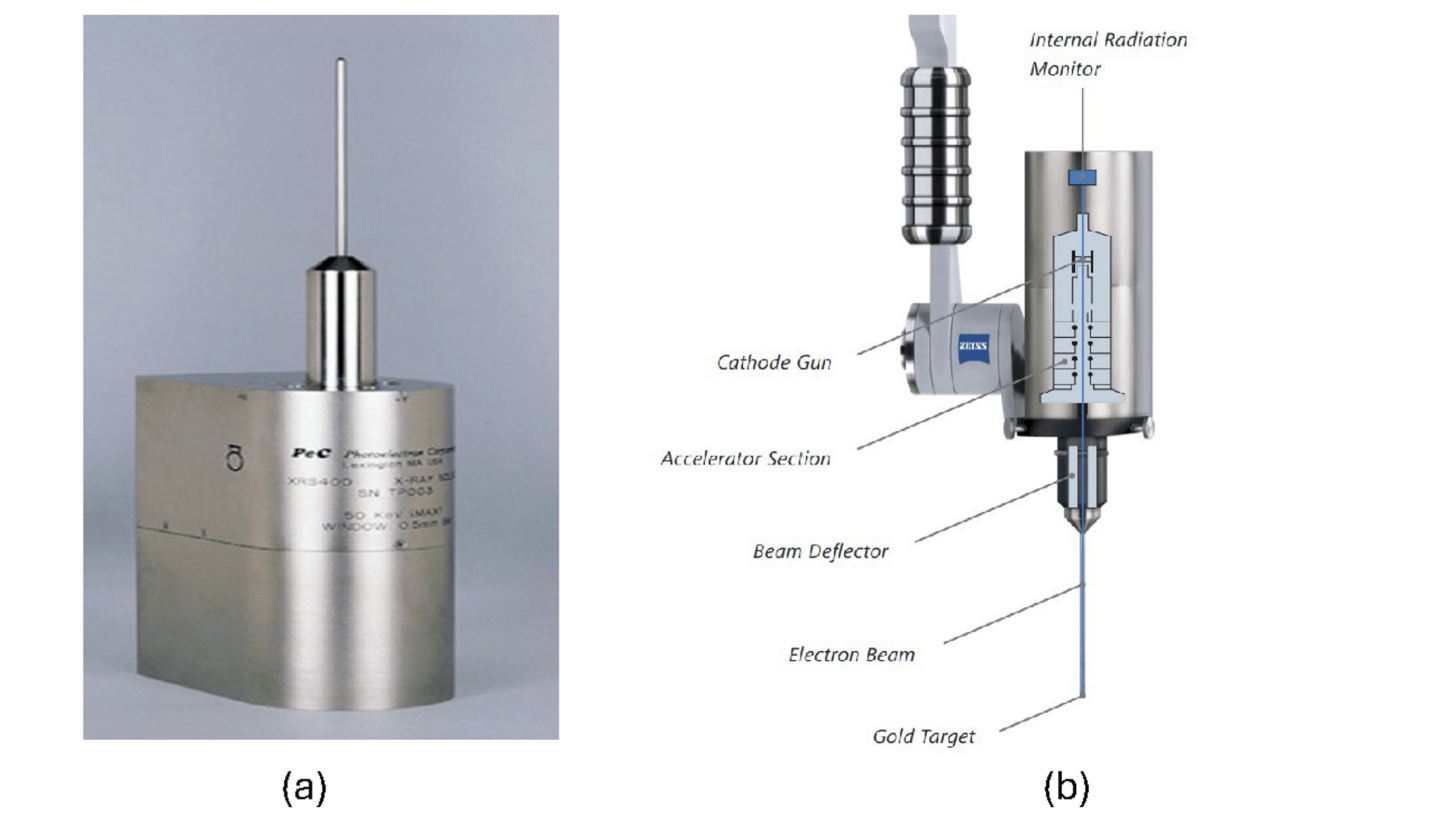
a) X-ray source (XRS). (b) Schematic of the electronic components of an XRS, depicted mounted on the arm manipulator. The XRS source standing up on the base of its housing is shown bare, i.e., without an applicator attached. The electronic generation of the X-rays in the XRS is described in the text (Figures from Zeiss [4] with permission).

A close-up view of the XRS without any applicator attached is shown in Figure 2a, and a schematic diagram of the XRS electronic components used to produce the X-rays is shown in Figure 2b. To generate the X-rays for treatment, electrons from the cathode are accelerated and guided down the drift tube by deflector coils towards the tip of the probe, where they hit a thin gold target producing the low-energy 50 kV X-rays. Precise centering of the electron beam towards the gold target is essential for the generation of an isotropic X-ray field from the tip of the probe. The Internal Radiation Monitor (IRM) is a calibrated photodiode at the back of the XRS housing with the function of monitoring the instantaneous output of the XRS, by detecting photons scattered backwards from the target [1,4,5]. 

To prepare the system for treatment in the OR, the XRS is mounted securely at the end of the semi-robotic arm (Figure 1b) and connected with a long cable running through the arm to the control console. A sterilized applicator is attached to the XRS, and a sterile drape is then placed over the arm [6]. The surgeon manipulates the arm to bring the XRS to the treatment site with six degrees of freedom, i.e., to any position and angulation into the patient as needed. The floor stand supporting the semi-robotic arm is locked in place once the source and applicator are in treatment position. The user enters into the control console details of the treatment to be delivered, i.e., patient data, the site to be treated, prescribed dose and depth, and the applicator selected for the case. With all required QA tests performed successfully ahead of time, the user can now initiate treatment. The control console displays graphically throughout the treatment the current X-ray output and the centering of the electron beam. It also displays numerically the cumulative dose delivered at all times, and the time remaining for treatment completion. Detailed data of the radiation delivery and results of the preliminary QA tests are saved on the console computer and can be exported afterwards for documentation.

Quality assurance

**Figure 3:**
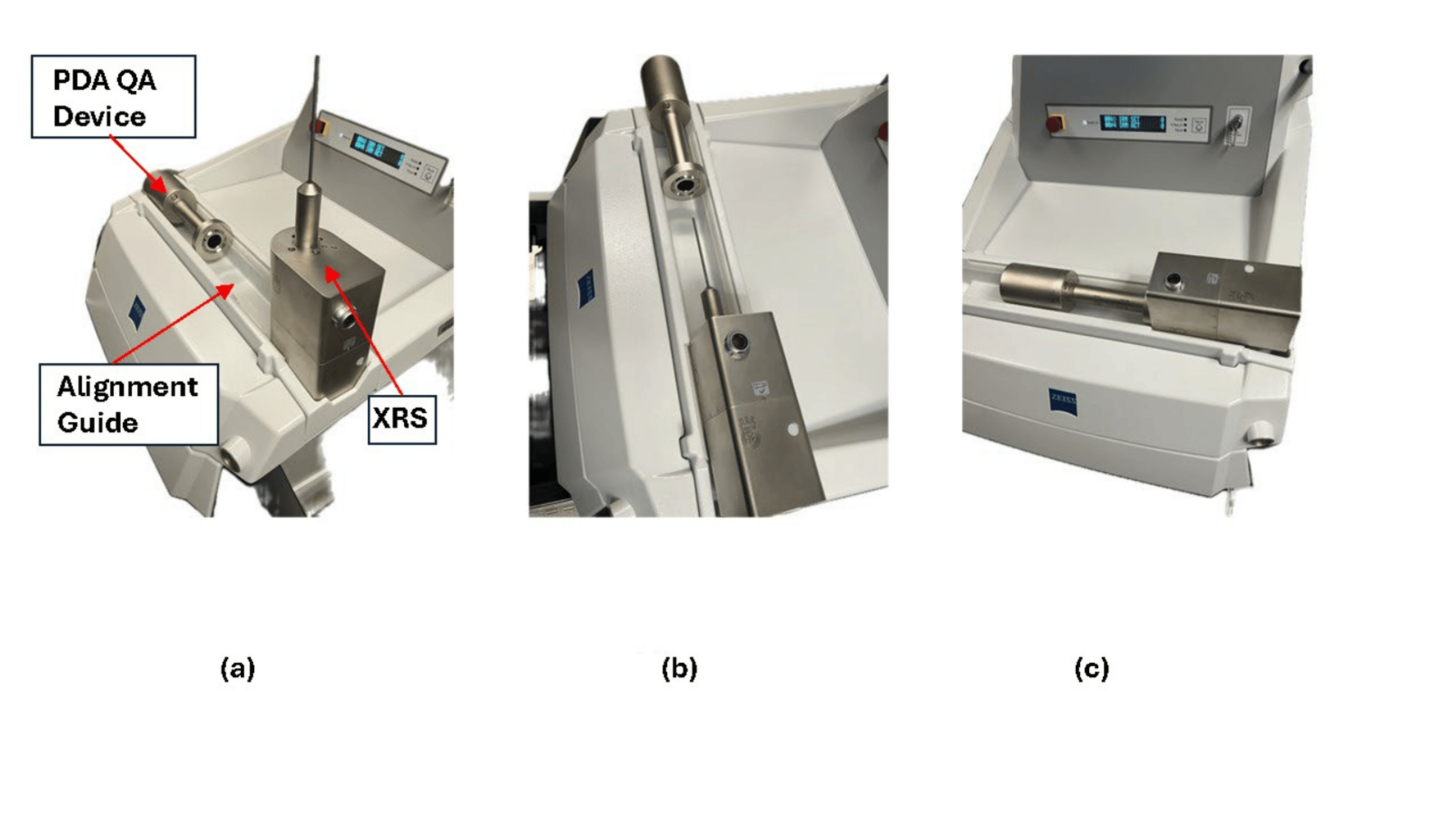
Mounting a QA device over the XRS unit for testing can be accomplished safely using the customized alignment guide of the console work area. The QA device can be mounted over the XRS precisely and without risk of damage to the probe using the customized alignment guide. (a) The QA tool is positioned horizontally in the guide. (b) The XRS is similarly placed on the guide. (c) The tool is slid towards the XRS, thus positioning the XRS probe centered inside the tool. The XRS and QA tool can then be placed upright on the work area and properly connected to the console for testing. (Figures are original from the authors.)

To deliver an IORT treatment to a patient, the Intrabeam system requires the successful execution of two pre-operative daily QA tests [6,7]. The first mandatory daily test is the isotropy check to determine whether the radiation field emitted from the target at the tip of the XRS probe generates a uniform angular distribution. In case the isotropy is found out of tolerance due to a probe misalignment, the procedure explained below can help correct it. If there were other less common system-related factors beyond a probe misalignment, they may require system servicing. For this isotropy test, a dedicated Photo-Diode-Array (PDA) shielded QA device provided by the manufacturer can be mounted precisely over the XRS using a customized plastic alignment guide in the working area of the console (Figure 3).

**Figure 4:**
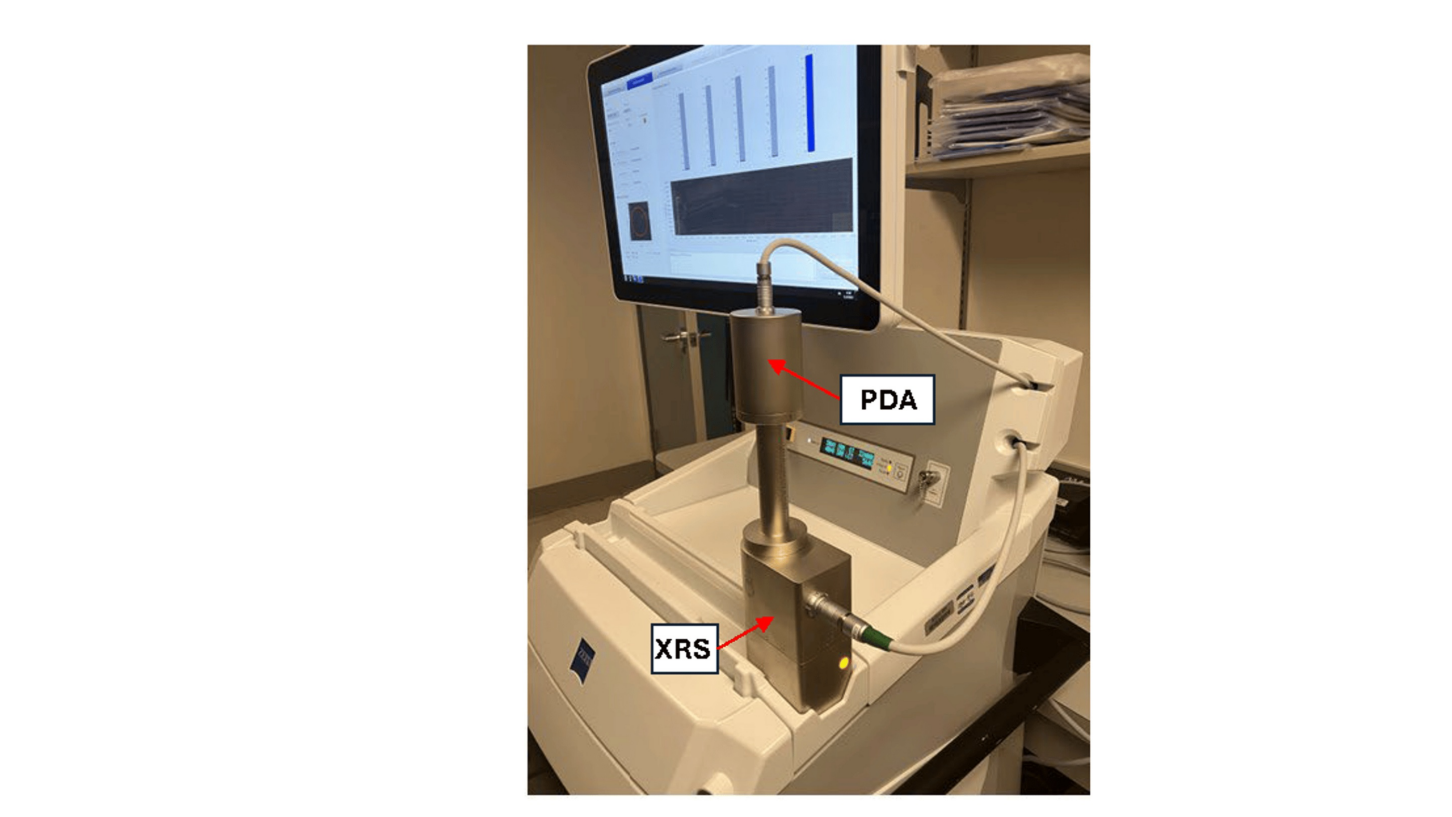
The Photo-Diode-Array (PDA) device is a shielded QA attachment used to measure isotropy of the radiation field around the XRS probe tip. The PDA is shown mounted on the XRS, positioned upright in the work area and connected to the console. The PDA uses five photodiodes equidistant from the electron target in the probe to determine the isotropy of the X-ray radiation field generated. The XRS and the QA devices are connected to the console for power and control. The PDA tool is also used to adjust electronically the direction of the electron beam towards the probe tip whenever necessary. (Figure is original from the authors.)

Having the PDA tool mounted over the XRS in this manner positions the tip of the probe, i.e., the gold target, in a fixed position near the center of the QA device. The PDA and the XRS are each connected to the console for power and control (Figure 4). To determine the isotropy of the X-ray field emitted from the XRS target, the PDA device contains five diodes equidistant from the ideal central location of the probe tip [1,5]. Four of these diodes lie along the plane that is perpendicular to the axis of the probe (i.e., along +-X and +-Y directions) and intersects the target at the tip of the probe, while the fifth diode lies along the longitudinal axis of the probe (+Z axis). This arrangement of the diodes makes the PDA sensitive to changes in the low-energy X-ray field isotropy stemming from sub-millimeter deviations of the XRS target centricity. In addition, the PDA device is utilized in a separate QA routine for re-steering the electron beam whenever a probe alignment is performed, as explained below.

**Figure 5:**
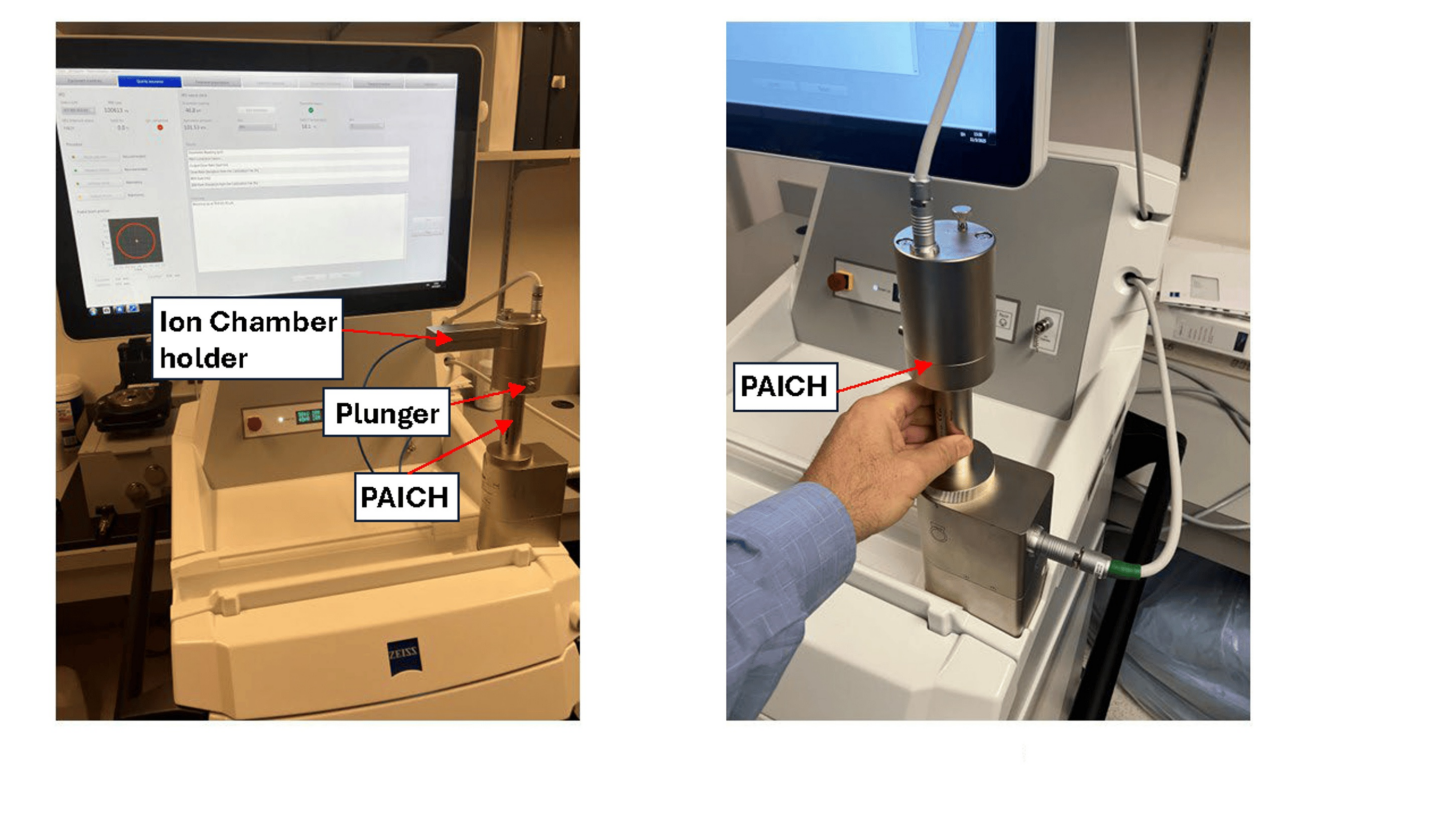
a) The Probe-Adjustment Ion-Chamber-Holder (PAICH) QA device is a second shielded attachment, designed for measuring the XRS output using an ion chamber. b) The PAICH is also utilized to measure, and correct if needed, the eccentricity of the probe tip which requires the user to rotate the device manually around the stationary XRS probe. (a) The output of the XRS is measured with a small parallel-plate ion chamber inserted in the opening near the PAICH top. (b) The PAICH is also used to determine probe eccentricity by manually rotating it 360 degrees around the XRS probe. It also allows the user to adjust the probe mechanically using the plunger on its side whenever it is required. (Figures are original from the authors.)

The second mandatory daily test is the output check, realized only after beam isotropy has been confirmed to be within tolerance. The output test measures the daily X-ray output using a second dedicated shielded QA device, the Probe-Adjustment Ion-Chamber-Holder (PAICH) (Figure 5a). A small calibrated parallel-plate ion chamber secured to a holder is inserted precisely into the slot near the top of the PAICH, and connected to a calibrated electrometer in the control console cart to determine the daily output of the X-ray source and to calibrate the internal radiation monitor (IRM) [1,5]. Output measurements are corrected for temperature and pressure variations with built-in calibrated instruments. The X-ray output obtained is used later on during treatment preparation to determine the irradiation time necessary for accurate dose delivery, depending on the prescribed dose, reference depth, and specific applicator selected for the case [5,7].

In case the initial isotropy measured is not within tolerance due to the XRS probe tip being out of alignment, a probe adjustment (or “XRS tip adjustment”) procedure becomes immediately mandatory [6,7] to re-center the probe. However, if an isotropy failure may arise for reasons other than a misaligned probe, the XRS would require service. The probe adjustment procedure is performed utilizing the PAICH device mounted on the XRS by manually rotating the device around the stationary XRS probe (Figure 5b) while the system monitors continuously the position of the probe tip. A continuous dynamic monitoring of probe eccentricity during this procedure increases the susceptibility of measurement to any motion-induced artifacts. The PAICH device uses a light source and a photodiode [1,5] to determine centering of the probe tip as the device is rotated a full turn. The PAICH also allows very fine (sub-millimeter) mechanical realignment of the probe by using the hammer or "plunger" on the side of the device to exert very small pushes of the probe in the necessary direction (Figure 5b). This procedure to correct probe misalignments is discussed in the next section, where we also suggest a modification to improve efficiency.

**Figure 6:**
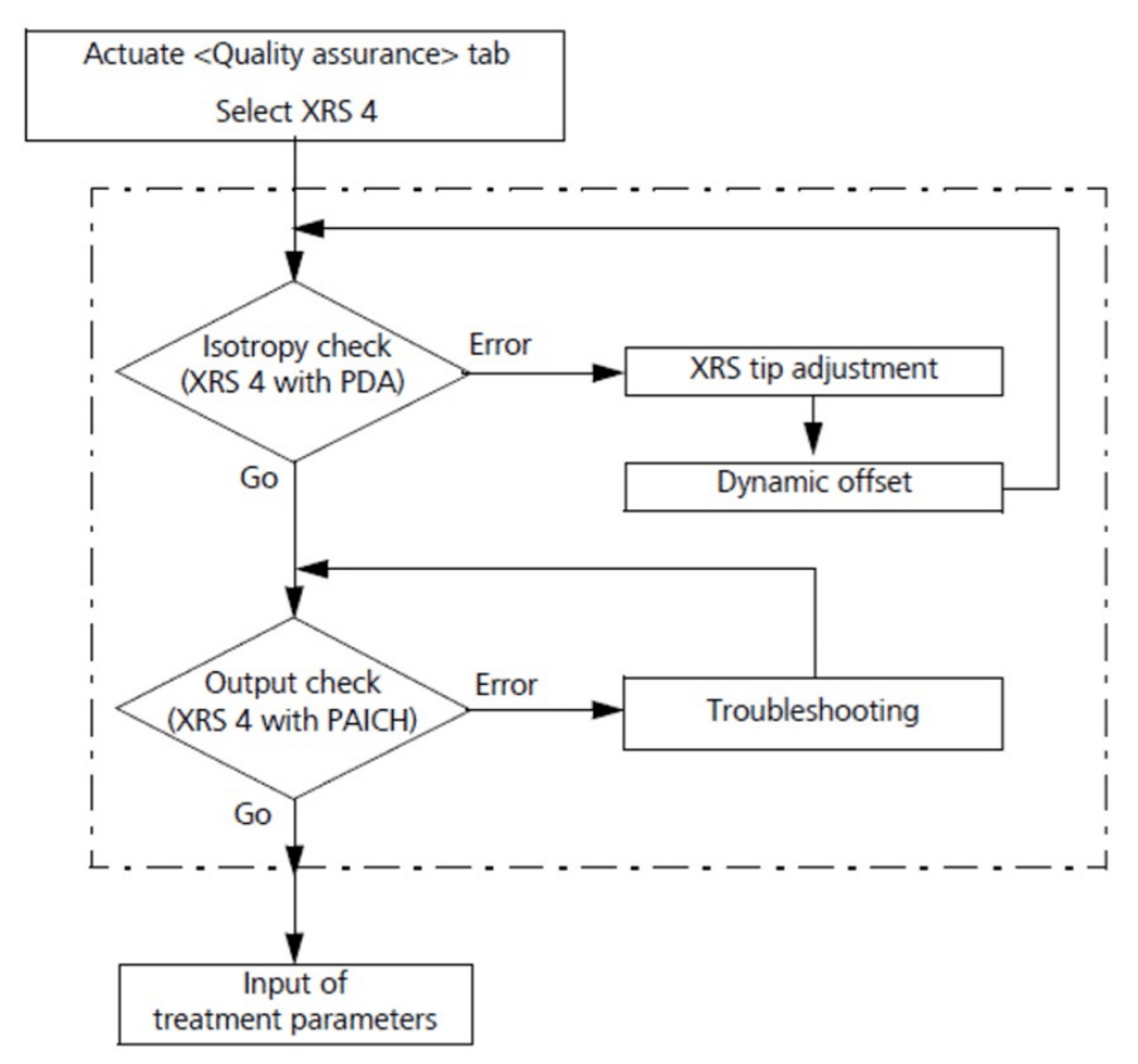
Daily Intrabeam Quality Assurance workflow to assure that X-ray isotropy is acceptable and to obtain daily XRS output. Whenever the isotropy check (using the PDA) fails, the user must execute the probe tip adjustment (using the plunger of the PAICH). After mechanical adjustment of the probe, the automatic dynamic offset steering procedure is performed, and then the isotropy test is repeated. If the isotropy test is now successful, the user moves on to the output test using the ion chamber inserted in the PAICH. If the isotropy is still not acceptable, the user must repeat the probe adjustment loop again (Figure from Zeiss [6] with permission).

A flowchart of the daily QA procedures for the Intrabeam from the user's guide is shown in Figure 6. The initial isotropy check must be acceptable before proceeding to the Output check. If isotropy is not acceptable, a mechanical adjustment of the probe tip must be realized successfully, after which the operator runs the dynamic offsets automatic procedure, which uses the beam deflector coils of the XRS to steer the electrons down the center of the realigned probe towards the gold target at the tip of the probe [1,5]. Once this steering is completed, the system requires the user to run the isotropy test again to confirm it remains within tolerance following the mechanical and electronic adjustments. This loop of consecutive checks (initial isotropy check, tip adjustment, electronic steering, mandatory final isotropy check) must be repeated until the isotropy is acceptable before proceeding to the Output check, as the QA workflow in Figure 6 shows. Based on our own institutional experience, in this sequence of QA steps, the procedure to adjust the probe of an XRS commonly takes the longest time to realize.

Efficient probe adjustment

An IORT treatment with the Intrabeam system cannot proceed while the probe of the X-ray source is out of alignment, i.e., until the probe tip is adjusted to the eccentricity tolerance of <0.1 mm. The probe adjustment procedure determines the extent and direction of misalignment of the XRS probe detected with the isotropy test and provides the means to correct this deviation. The user’s guide of the Intrabeam system [6] directs the operator to mount the PAICH QA device over the XRS housing, as explained in the previous section, and manually rotate the PAICH a full turn around the stationary XRS probe (Figure 5b) while the system continuously monitors the eccentricity of the probe. If the total deviation of the probe tip from the ideal center obtained after a full turn exceeds the specified tolerance, the test is deemed to have failed, and the probe must be adjusted. Therefore, as directed, to be successful, this procedure requires that eccentricity remains within tolerance even while the device is being manually rotated around the XRS probe. This requirement is not per se a necessary condition for failure of the eccentricity measurement, because such faulty readings may arise not from probe misalignment, but from extraneous motion artifacts introduced during handling of the PAICH tool having no relevance during time of treatment delivery, possibly due to factors such as 1) movement artifacts from uneven handling of the PAICH tool, 2) rotation irregularities caused by friction between the moving metal surfaces in contact (the base of the PAICH against the front plate of the XRS housing), 3) wobbling or vibrations of the PAICH device while it is made to turn, etc. Thus, artifacts such as these are unrelated to probe alignment and could lead to false-negative results in probe eccentricity checks.

In this report, we suggest a modification of this probe adjustment procedure to avoid delays from such false-negative results, which could contribute to the general objective of reducing overall IORT treatment times [8]. As an alternative to requiring that measurements of eccentricity remain within tolerance throughout a full rotation of the PAICH around the XRS probe, centering deviations considered are limited to discrete or “static” angular positions of the PAICH, at which the user completely releases their grip on the PAICH device. That is, any “dynamic” eccentricity readings detected by the system while the PAICH is being rotated are disregarded. Final acceptance of probe eccentricity remains contingent upon passing manufacturer-required isotropy and other QA checks, and all acceptance criteria remain unchanged.

Based on our practical experience, an initial full rotation can be done, at first, at static angles every 10 to 15 degrees to detect the angular sector, out of the full rotation, observed to include the larger positive deviations. This can be followed by a finer static sampling, limited now to the angular sector first detected. After having identified the direction of the largest positive deflection (the “max” in the user's guide) and negative deflection (the “min”) at the opposite direction from the static angles sampled, the probe is then adjusted in the required direction using the plunger, following the system user’s guide [6]. Specifically, the user plunges the probe in the direction of maximum deviation, aiming to decrease the reading by about half the total deviation, i.e., by ½ of (max - min), to bring the probe closer to the ideal center. After this adjustment, a confirmatory re-sampling of eccentricity deviations is performed, again only at static angles throughout the 360-degree range. If necessary, the probe adjustment step would be repeated in the newly detected static direction of maximum deviation until the eccentricity is acceptable.

Once a probe eccentricity of <0.1 mm is achieved following a full rotation sampling of static angles, the probe is assumed to be aligned by the criterion established by the system. The user can then restart the probe adjustment test in the console QA routine by zeroing the eccentricity to clear any previous values of deviation and accept the test without any further rotation of the device. After the probe adjustment is accepted, the user is required to initiate the automatic dynamic offset procedure [6] using the PDA device, which establishes the electronic steering for the system to direct the electron beam towards the mechanically re-centered XRS target at the tip of the probe. By design, the Intrabeam system requires repeating the isotropy QA test after every probe adjustment run [6], whether the latter is realized by following the manufacturer’s recommended “dynamic” procedure or by the modified “static” approach suggested here. Thus, with the requirement of a successful final isotropy QA check, the system guarantees that the probe eccentricity is indeed within tolerance, and thus the proposed modification does not compromise system safety or dosimetric integrity. Once all required QA tests have been completed successfully, the unit is ready to proceed with preparations for delivery of the IORT treatment.

## Discussion

Utilization of the Intrabeam system for IORT treatments requires the successful execution of two mandatory daily QA pre-operative tests, the isotropy check, and the output check. These can usually be completed by the physics team ahead of time in about 10 minutes. However, if the initial isotropy test fails because of a misalignment of the XRS probe tip, a probe adjustment procedure becomes necessary to bring the tip of the probe back closer to its ideal center. Completing this procedure can take significantly more time than the two initial ones because, unlike the first two "hands-off" checks, the probe adjustment procedure involves an interplay of the operator manipulating the PAICH tool around the XRS with the system continuously monitoring deviations from the ideal center of the probe in real time.

Given its "hands-on" nature, negative results of the probe adjustment procedure can be caused by factors that have no relevance at the time of delivering an IORT treatment appropriately, such as irregularities in the rotation of the PAICH due to an operator’s irregular grasp or movement of the device, the condition of the metal surfaces in contact as the PAICH is rotated, vibrations or movements of the console, tension of the cable connecting the PAICH to the controller console, environmental conditions in a crowded and busy OR setting, etc. In our experience, the very tight tolerance for XRS target eccentricity of <0.1 mm required by the Intrabeam system to ensure that the low-energy X-ray beam will be isotropic to treat a target volume appropriately can make adjusting the position of the target of the probe very challenging. Often, multiple repetitions of the procedure are needed to achieve acceptable eccentricity. Reducing the incidence of false-negative results could help significantly improve efficiency in IORT treatment preparation. The system tolerances and acceptance criteria remain unchanged under the proposed refinement in workflow efficiency.

An XRS probe is at its greatest risk of suffering misalignment in a busy or crowded OR while handled by the medical staff in instances such as (1) accidental physical impact of the unprotected XRS probe during equipment preparation; (2) attaching an applicator to the XRS source by a surgeon or nurse; (3) while draping the semi-robotic arm for sterility; (4) during insertion of the applicator, e.g., into a lumpectomy cavity; (5) while suturing the applicator in place for treatment, etc. When a misalignment of the XRS probe occurs in the OR as the medical team completes preparations for an IORT treatment, it is important to bring the probe back to its center as quickly as possible. The probe adjustment procedure itself can take 20 to 30 minutes or even longer to complete when following the procedure indicated in the system user’s guide, based on our experience and as reported anecdotally by other experienced users. In our experience with the modified methodology proposed here, this procedure can often be completed in about 10 minutes. These time estimates are based on operational experience rather than formally measured or statistically analyzed data. Shortening the time to complete all the QA tests required in such a scenario would be advantageous to the medical teams involved, as well as limiting any additional time the patient is kept under general anesthesia.

Since all daily QA tests of the Intrabeam system are performed using a bare XRS, i.e., before the selected applicator is attached to the XRS, any efficiency improvement in completing the daily QAs for an IORT delivery could benefit treatments for all body sites, independent of the type of applicator selected for treatment.

Reducing the number and length of repetitions of the probe realignment procedure performed on an XRS may help extend its performance, as repeated mechanical stress from excessive plunging may weaken the probe of the XRS [5]. Finally, it should be noted that, according to the manufacturer, in cases of excessive probe bending (for instance, due to a significant impact to the unprotected XRS probe), use of the plunger of the PAICH tool may not be adequate to bring the probe back into proper alignment, and therefore the present modification to the adjustment procedure would certainly not be able to re-center the probe either. In such cases, the XRS source must be returned to the vendor for repair or replacement.

## Conclusions

Finding that the probe of the X-ray source has been mechanically compromised in the OR just before commencing an IORT treatment with the Intrabeam system will require the user to successfully conduct a series of QA tests to correct the probe misalignment as quickly as possible. Among these QA steps, the probe realignment procedure usually takes the longest time to accomplish. However, the "hands-off" modified probe adjustment method may only take about 10 minutes to complete, reducing the total QA duration from about 35 to 45 minutes or more, down to about 25 minutes.

A more efficient execution of the required pre-operative checks could potentially benefit the patient by shortening any additional time under general anesthesia, as well as the OR medical teams involved in this treatment modality. This potential benefit is inferred from reduced QA duration rather than from directly measured clinical outcome.
